# Facile preparation of polyolefin-based amphiphilic graft copolymer fibers by combination of photoinduced graft copolymerization and electrospinning

**DOI:** 10.55730/1300-0527.3562

**Published:** 2023-04-12

**Authors:** Çağatay ALTINKÖK

**Affiliations:** 1 Department of Chemistry, Faculty of Science and Letters, İstanbul Technical University, İstanbul, Turkey

**Keywords:** Amphiphilic, chlorinated polypropylene, free-radical polymerization, poly(oligoethylene glycol methacrylate)

## Abstract

A novel amphiphilic graft copolymer possessing polypropylene (PP) main chain and poly(oligoethylene glycol methacrylate) (POEGMA) pendant units was synthesized starting from chlorinated polypropylene (PP-Cl), and characterized. PP-Cl produced macroradicals at chlorine bounded carbon atoms by visible light irradiation in the presence of dimanganese decacarbonyl [Mn_2_(CO)_10_] and initiated the free-radical photopolymerization of an acrylate monomer, namely oligoethylene glycol methacrylate (OEGMA). Furthermore, fiber formation ability of PP-*g*-POEGMA was tested by electrospinning technique. The chemical structure and some features of the corresponding amphiphilic graft copolymer PP-*g*-POEGMA was characterized by implementing spectral (FT-IR, ^1^H-NMR), chromatographic (GPC), morphological (SEM), water wettability (WCA), and thermal (TGA) analyses. It was clear from the SEM results that the average diameter of the obtained microfibers decreased with the incorporation of POEGMA segments onto the PP-Cl main chain. Based on WCA measurements, PP-*g*-POEGMA was determined as more wettable than PP-Cl due to its hydrophilic POEGMA building blocks. This facile procedure could be utilized to achieve the amphiphilic commercial polymers for potential bioapplications such as drug delivery.

## 1. Introduction

Polyolefins, which are formed by the polymerization of olefins such as propylene and ethylene, are the most widely used polymers in the world due to their low cost, good recyclability, and mechanical properties [1]. Since they have high resistance to chemicals and wide range of mechanical properties, they are frequently preferred in food packaging, automotive, construction, health, and similar sectors [2–4]. Despite their unique properties in the field, they have nonpolar carbon and hydrogen atoms in their main skeleton, and their poor compatibility with other materials cause some limitations [5]. These limitations can be overcome by chemical modifications of polyolefins and, at the same time, by improving material properties (e.g., processability, chemical robustness, and mechanical strength) [6–9]. Therefore, functionalization of polyolefins has been a research subject for many decades. On the other hand, postpolymerization processes can cause other problems such as degradation of their backbone. Thus, the studies to develop new chemistry that can address the challenge of preparing functionalized polyolefins become a fundamental need. The halogenation process, which is one of the modification methods of polyolefins, not only provides a change of polarity by introducing halogen atoms (fluorine, chlorine, bromine, or iodine) to their nonpolar backbones but also creates active sites for chemical reactions [10]. Unfortunately, PP is also the most difficult to functionalize by existing processes and only a Ziegler-Natta polymerization can be used to prepare PP with desirable properties. On the other hand, chlorination of PP with chlorinated solvents or materials under suitable conditions is a good example of the functionalization processes [11]. Thus, the polarity of PP-Cl can be improved its adhesion, stability, and wettability properties compared to polypropylene, and also PP-Cl can be modified by using the chlorine atoms on the polymer chains [12]. Grafting approaches applied to PP-Cl with many chemical routes have been reported in the literature. One of the most common events of these modifications is maleic acid grafting in the presence of an initiator. At the same time, various polyolefin-based graft copolymers were synthesized by the atom transfer radical polymerization (ATRP) and copper catalyzed azide alkyne cycloaddition (CuAAC) using the chlorine atoms of PP-Cl [13–16].

**Figure 1:**
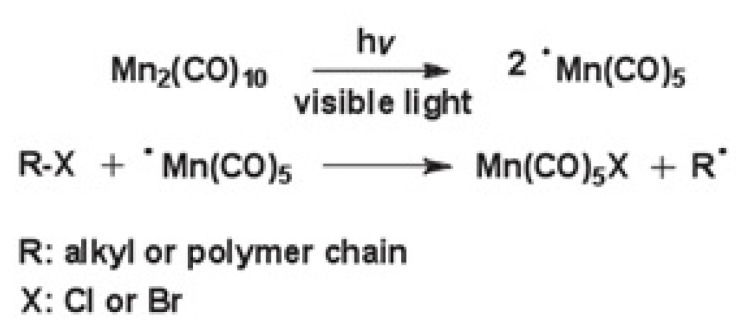
Photoinduced free-radical generation from halides by using Mn_2_(CO)_10_.

Many derivatives of organometallic compounds are photochemically active and thus can be used as initiators for the polymerization of vinyl monomers at certain wavelengths [17]. Dimanganese decacarbonyl (Mn_2_(CO)_10_) compound, which is one of the organometallic derivatives, has a weak Mn-Mn bond and absorbs light in the range of almost 460–380 nm, forming the · Mn(CO)_5_ metalloradical with good quantum efficiency. One of the most widely used methods for photochemically generated free radical using Mn_2_(CO)_10_ is to produce a carbon-centered radical by abstraction halogen from an alkyl halide (Figure 1). Although the use of Mn_2_(CO)_10_ in light-induced polymerization processes has not been fully controlled, it has been reported in the literature as an ideal method for obtaining block and graft polymers [18–20].

Electrospinning is an efficient and simple process widely used for producing of polymeric fibers from soluble and fusible natural and synthetic polymers with diameters ranging from 2 nanometers to several micrometers [21]. Compared to fibers produced by other methods, those produced by electrospinning have been recently preferred more in areas such as nanocatalysis, tissue engineering, protective clothing, filtration, biomedical, pharmaceutical, and optical electronics due to their small pore structures and high surface areas [22–26]. Some basic parameters affecting the electrospinning are; (1) process parameters (feed rate, accelerating voltage, tip-to-target distance, and needle diameter), (2) solution parameters (solvent, molecular weight, viscosity, polymer concentration, and solution conductivity), and (3) environmental parameters (temperature, pressure, and relativity humidity). By optimizing these parameters, it is possible to obtain polymeric fibers in desired scales.

Polyolefin derivatives such as polyethylene and polypropylene require elevated temperatures, additional experimental setups, as well as nonpolar solvents during electrospinning bringing many disadvantages. Recently, there are many different approaches in the literature with the purpose of enhancing the electrospinnability of polyolefin backbone to overcome these drawbacks, including incorporation of various functional groups to the main chain or grafting different types of polymers [27, 28].

In the light of the all of the aforementioned information, herein, the microfibers of amphiphilic graft copolymer possessing PP-Cl and POEGMA were easily achieved via sequential Mn_2_(CO)_10_ photochemistry and electrospinning process. The resultant microfibers were characterized in detail by spectral, chromatographic, microscopic, water wettability and thermal analyses, and compared with their precursors at various stages.

## 2. Experimental part

### 2.1. Materials

PP-Cl (chlorine mass fraction: 29%–32% (m/m) was supplied from Mark Zhang Shanghai Sunking Industry Incorporation (Shanghai, China). The number average molecular weight (*M*_n_) of PP-Cl was found to be 135,000 g mol^−1^ by GPC analysis in our laboratory. Dimanganese decacarbonyl (98%, (Mn_2_(CO)_10_)) was purchased from Sigma-Aldrich and purified by sublimation before use. POEGMA (*M*_n_ = 360 g mol^−1^) was supplied from Merck and purified by passing through a column filled with neutral alumina before use. Tetrahydrofuran and methanol were purchased and used as stored with drying agent.

### 2.2. Instrumentation

Proton nuclear magnetic resonance (^1^H-NMR, 500 MHz, in CDCl_3_-*d*_6_ solvent using tetramethylsilane internal reference) analyses of the samples were conducted with an Agilent VNMRS 500 instrument. Fourier transform infrared (FT-IR) spectroscopy was done at a scanning range of 4000–500 cm^−1^ with 16 scan numbers via a Cary 630 FTIR (Agilent Technologies) and utilized to determine the specific functional groups. Gel permeation chromatography (GPC) (Agilent 1100, THF eluent at 0.3 mL min^−1^ flow rate at 30 °C, calibrated with linear polystyrene standards) analysis was performed to determine the molecular weight of the polymers. The surface morphologies, average fiber diameters, and histograms of the samples were screened with SEM (Zeiss Evo LS 10) and Image J software. A NanoLinker Contact Angle Measurement System (CA-500A) equipped with a Hamilton microsyringe containing a 22-gauge needle was used to conduct the WCA measurements. TGA analyses were conducted under nitrogen atmosphere at the range of 25–600 °C and 10 °C/min heating rate by a TGA 8000 (Perkin Elmer).

### 2.3. Procedure for the preparation of amphiphilic graft copolymer (PP-g-POEGMA)

First, PP-Cl (500 mg, 3.7 × 10^−3^ mmol) was put into a Pyrex tube equipped with a magnetic stirring bar and allowed to dissolve in the freshly distilled THF (3 mL) for 2 h. To this solution, OEGMA (0.8 mL, 2.5 × 10^−3^ mol) and Mn_2_(CO)_10_ (9.7 mg, 2.4 × 10^−2^ mmol) were added and purged with nitrogen for 5 min before irradiation by a photoreactor equipped with six lamps (Philips TL-D 18 W) emitting light nominally at 400–500 nm at room temperature. After 3 h, the resulting polymer was precipitated into cold methanol and then dried under vacuum overnight.

### 2.4.Electrospinning procedure

After the desired block copolymer was obtained, PP-*g*-POEGMA and PP-Cl fibers having bead-free morphologies were fabricated with the same electrospinning parameters. Briefly, 200 mg of pristine and modified polymers were separately dissolved in 1 mL of THF and individually loaded into disposable plastic syringes (1 mL) equipped with a pump which is on the opposite side of the aluminum collector. Typical electrospinning parameters were as follows: feeding ratio = 1.3 mL/h, applied voltage = 15 kV and tip-to-collector distance = 17 cm. Electrospinning process for both samples was carried out at 41 °C and 51% relative humidity for 30 min.

### 2.5. Water contact angle (WCA) measurements

The water wettability behavior of the electrospun microfibers was evaluated with the sessile drop method. The WCA of the microfibers deposited on glass slides were measured with 5 μL of ultrapure water droplet using a Contact Angle Analyzer equipped with programmable syringe pump, microsyringe, stainless steel needle and conventional digital camera working at 22–23 °C (laboratory temperature).

## 3. Results and discussion

**Figure 2:**
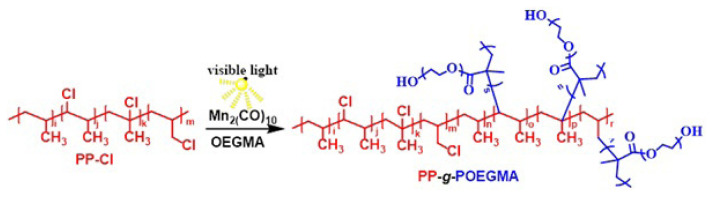
Synthetic approach for preparing PP-*g*-POEGMA.

Amphiphilic copolymers get great attention in many applications due to the wide variety of compositions, morphologies and properties designed to obtain these materials [21, 29]. Generally, macromolecules containing hydrophilic segments, such as poly(ethylene glycol) (PEG) derivatives, are of interest because of their biocompatibility and ability to self-assemble into higher-order structures [30, 31]. In this respect, we intended to prepare new amphiphilic polyolefin derivative by grafting POEGMA pendant units on PP-Cl main chain by free-radical photopolymerization in the presence of Mn_2_(CO)_10_ in one step. The synthesis of the amphiphilic graft copolymer was conducted as depicted in Figure 2.

**Figure 3:**
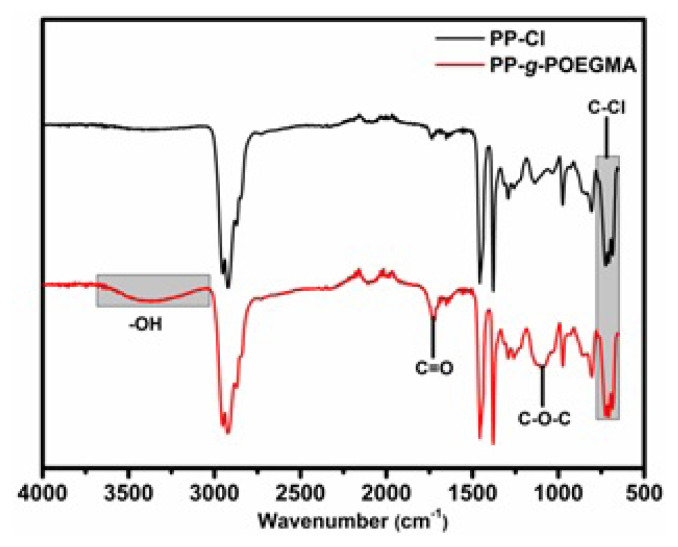
Comparative FT-IR spectra for PP-Cl and PP-*g*-POEGMA.

The chemical structures of PP-Cl and PP-*g*-POEGMA were identified by FT-IR with ATR equipment (Figure 3). The characteristic C-H peaks determined at 2930, 1480, and 1390 cm^−1^ and multiple C-Cl peaks that appeared at 657, 725, 757 cm^−1^ verified the structure of PP-Cl. In the spectrum of PP-*g*-POEGMA, the peaks belonging to PP-Cl main chain as well as -OH, C=O, and C-O-C peaks of POEGMA building blocks that appeared at 3030–3700, 1720, and 1100 cm^−1^, respectively, indicated the successful graft copolymerization [1, 32, 33]. One can see that the C-Cl peaks of PP-Cl are still clearly detectable in PP-*g*-POEGMA spectrum.

**Figure 4:**
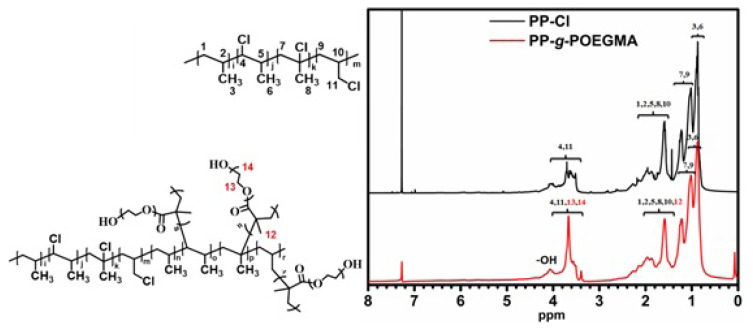
Comparative ^1^H-NMR spectra for PP-Cl and PP-*g*-POEGMA.

The success of photoinduced graft copolymerization was also evidenced by ^1^H-NMR spectroscopy, where the characteristic protons of both PP-Cl and PP-*g*-POEGMA segments were detected (Figure 4). The ^1^H-NMR spectrum of PP-*g*-POEGMA indicated characteristic peaks of precursors. In the case of PP-Cl spectrum, the signals detected between 1.71 and 2.81 ppm corresponded to -CH_2_ and -CH protons (**1**–**3** and **5**–**10**). Moreover, the signals determined at 3.55 and 3.68 ppm were associated with -CH_2_-Cl and –CH-Cl protons (**4** and **11**), respectively [32, 34]. In the spectrum of PP-*g*-POEGMA, the appearance of -OH proton signal at 4.10 ppm and the overlapped peaks with PP-Cl such as –CH_3_, -O-CH_2_, and -CH_2_ protons that should normally be seen at around 1.85 and 3.65 ppm (**12**, **13**, and **14**) as well as other remained characteristic proton signals of PP-Cl were great evidences for indicating the success of photoinduced graft copolymerization conducted [33].

**Figure 5:**
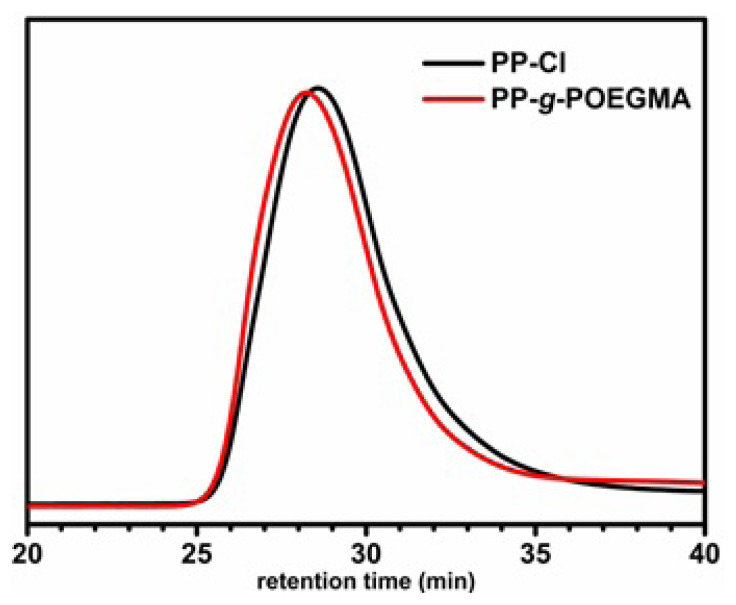
Overlaid GPC traces of PP-Cl and PP-*g*-POEGMA determined according to PS standards.

The molecular weight characteristics of the synthesized PP-*g*-POEGMA and pristine PP-Cl were determined by GPC analysis (Figure 5). As can be seen from GPC traces that displayed unimodal nature proved the absence of any side reactions during the grafting process. Compared with the PP-Cl, the GPC trace of PP-*g*-POEGMA proved the success of grafting by appearing in the lower retention time. The molecular weight of PP-Cl was determined as 135,000 g mol^−1^ with the PDI value of 2.6, whereas these values were found to be 185,000 g mol^−1^ and 2.6 for PP-*g*-POEGMA.

**Figure 6:**
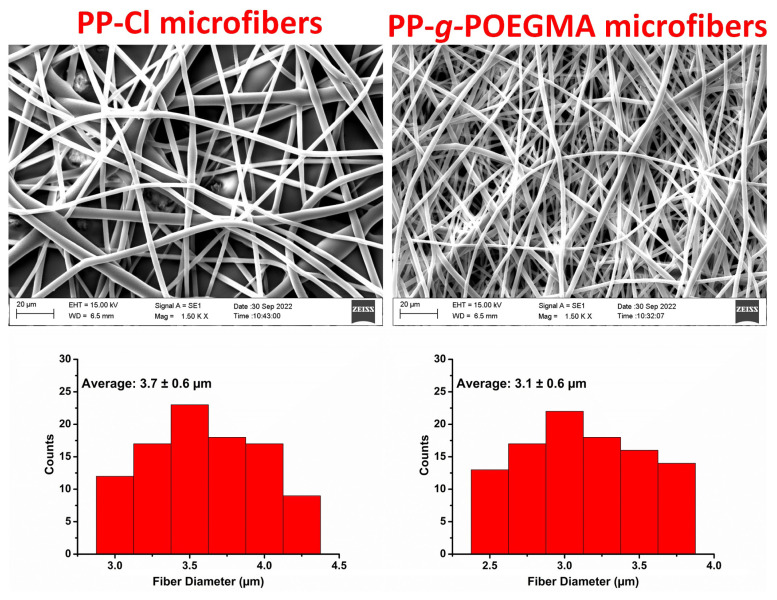
SEM images and fiber diameter distributions of PP-Cl and PP-*g*-POEGMA microfibers.

The achieved amphiphilic PP-*g*-POEGMA was electrospun and its morphologic features were compared with electrospun PP-Cl fibers. The SEM images of both PP-Cl and PP-*g*-POEGMA demonstrated the completely bead-free, microscale diameter, uniform and cylindrical morphologies (Figure 6). The electrospun PP-Cl fibers accumulated on the target collector has average diameter of about 3.7 ± 0.6 μm, whereas PP-*g*-POEGMA generated electrospun fibers having 3.1 ± 0.6 μm average diameter, captured at 2500× magnification. The reduction in the average fiber diameter after the graft copolymerization could be associated with the combined contributions of enhanced hydrophilicity [35, 36] and higher molecular weight leading to enhanced molecular interactions among the polymer chains during the electrospinning [37].

**Figure 7:**
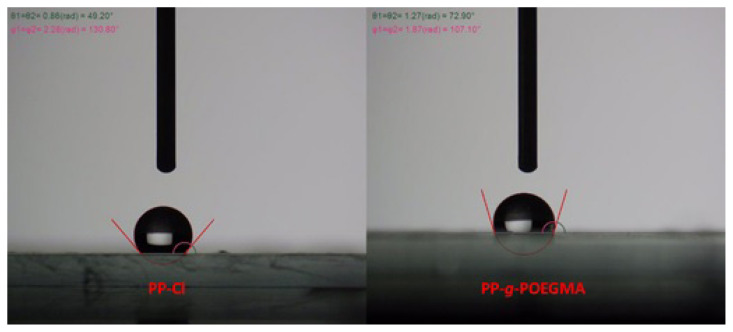
WCA analysis results of PP-Cl and PP-*g*-POEGMA electrospun fibers and static water droplet photographs on the samples captured by conventional digital camera.

One of the most important criteria affecting various properties of many materials is the wettability properties of that material [38]. Therefore, WCA analysis was carried out to provide information about the success of photoinduced graft copolymerization of the precursors and their wettability tendencies (Figure 7). The surface water wettability of the samples was determined by Sessile drop technique. As can be seen in this figure, the PP-Cl microfiber exhibited the hydrophobic behavior with the WCA value of 130.8° ± 2, when the PP-*g*-POEGMA microfibers were synthesized from PP-Cl by combination of photoinduced graft copolymerization and electrospinning, the surface wettability changed to a higher hydrophilicity decreasing WCA of 107.1° ± 2 due to the introduction of hydrophilic POEGMA pendant groups which strongly interacts with water drops.

**Figure 8:**
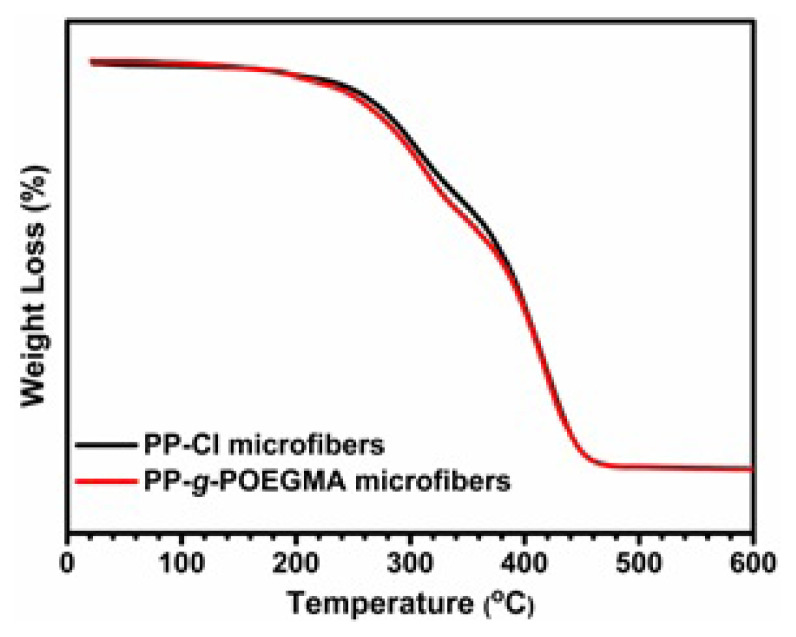
TGA curves of PP-Cl and PP-*g*-POEGMA electrospun microfibers.

The thermal properties of the final fibers were evaluated by TGA analysis, which is the generally accepted method indicating the decomposition/degradation steps of polymeric materials (Figure 8). As is known from the literature, the initial thermal decomposition of halogenated polymers usually starts with the dehydrochlorination process and finally ends with the respective chain scission [39]. In our case, the first thermal decomposition of PP-Cl microfibers initiated at 195 °C and the second step was observed at 415 °C owing to the dehydrochlorination and degradation of the unchlorinated PP backbone, respectively. On the other hand, PP-*g*-POEGMA microfibers showed a three-step thermal decomposition behavior. Unlike PP-Cl, the extra one thermal decomposition step determined at 345 °C resulted from POEGMA pendant groups attached to the PP main chain, as expected [40].

## 4. Conclusion

This work investigated the photoinduced graft copolymerization of a hydrophobic polyolefin PP-Cl and hydrophilic POEGMA to achieve PP-*g*-POEGMA in the presence of Mn_2_(CO)_10_ in one pot. Furthermore, electrospinnability of this obtained material was studied by comparing it with PP-Cl fibers. FT-IR, ^1^H-NMR, and GPC analyses clearly proved the formation of PP-*g*-POEGMA in the present concept. Electrospinning of PP-*g*-POEGMA gave rise to cylindrical, smooth, and bead-free microfibers with diameters of 3.1 ± 0.6 μm. The incorporation of POEGMA segments onto PP-Cl as a pendant group resulted in a decrease in average fiber diameter. Water contact angle measurements conducted on PP-Cl and PP-*g*-POEGMA revealed a change in wetting behavior from hydrophobicity to hydrophilicity after incorporation of POEGMA segments onto PP-Cl main chain. In summary, a combination of the photoinduced graft copolymerization and electrospinning provided a practicable approach for the preparation of different polymeric materials with desired features for many bioapplications.
